# Activation of the Nrf2/HO-1 Antioxidant Pathway Contributes to the Protective Effects of Lycium Barbarum Polysaccharides in the Rodent Retina after Ischemia-Reperfusion-Induced Damage

**DOI:** 10.1371/journal.pone.0084800

**Published:** 2014-01-06

**Authors:** Meihua He, Hong Pan, Raymond Chuen-Chung Chang, Kwok-Fai So, Nicholas C. Brecha, Mingliang Pu

**Affiliations:** 1 Department of Anatomy/Embryology, School of Basic Medical Sciences, Peking University, Beijing, China; 2 Key Laboratory on Machine Perception (Ministry of Education), Peking University, Beijing, China; 3 Key Laboratory for Visual Impairment and Restoration (Ministry of Education), Peking University, Beijing, China; 4 Department of Anatomy and Research Center of Heart, Brain, Hormone and Healthy Aging, LKS Faculty of Medicine, Pokfulam, Hong Kong, China; 5 The State Key Laboratory of Brain and Cognitive Sciences, The University of Hong Kong, Pokfulam, Hong Kong, China; 6 GHM Institute of CNS Regeneration, Jinan University, Guangzhou, China; 7 Department of Ophthalmology, The University of Hong Kong, Pokfulam, Hong Kong, China; 8 Department of Neurobiology, Department of Medicine, Jules Stein Eye Institute, CURE Digestive Diseases Research Center, David Geffen School of Medicine at Los Angeles, University of California Los Angeles, Los Angeles, California, United States of America; 9 Veterans Administration, Greater Los Angeles Health System, Los Angeles, California, United States of America; University of Pecs Medical School, Hungary

## Abstract

Lycium barbarum polysaccharides (LBP), extracts from the wolfberries, are protective to retina after ischemia-reperfusion (I/R). The antioxidant response element (ARE)–mediated antioxidant pathway plays an important role in maintaining the redox status of the retina. Heme oxygenase-1 (HO-1), combined with potent AREs in its promoter, is a highly effective therapeutic target for the protection against neurodegenerative diseases, including I/R-induced retinal damage. The aim of our present study was to investigate whether the protective effect of LBP after I/R damage was mediated via activation of the Nrf2/HO-1-antioxidant pathway in the retina. Retinal I/R was induced by an increase in intraocular pressure to 130 mm Hg for 60 minutes. Prior to the induction of ischemia, rats were orally treated with either vehicle (PBS) or LBP (1 mg/kg) once a day for 1 week. For specific experiments, zinc protoporphyrin (ZnPP, 20 mg/kg), an HO-1 inhibitor, was intraperitoneally administered at 24 h prior to ischemia. The protective effects of LBP were evaluated by quantifying ganglion cell and amacrine cell survival, and by measuring cell apoptosis in the retinal layers. In addition, HO-1 expression was examined using Western blotting and immunofluorescence analyses. Cytosolic and nuclear Nrf2 was measured using immunofluorescent staining. LBP treatment significantly increased Nrf2 nuclear accumulation and HO-1 expression in the retina after I/R injury. Increased apoptosis and a decrease in the number of viable cells were observed in the ganglion cell layer (GCL) and inner nuclear layer (INL) in the I/R retina, which were reversed by LBP treatment. The HO-1 inhibitor, ZnPP, diminished the LBP treatment-induced protective effects in the retina after I/R. Taken together, these results suggested that LBP partially exerted its beneficial neuroprotective effects via the activation of Nrf2 and an increase in HO-1 protein expression.

## Introduction

Retinal ischemia-reperfusion (I/R) injury is associated with many ocular diseases, including acute glaucoma and diabetic retinopathy [1], [2]. Interruption of the blood supply to an organ results in a wide variety of metabolic impairments, and the process of reperfusion itself is deleterious to injured cells due to the generation of free radicals and inflammatory cytokines [3]. Oxidative injury is one of the complications after retinal ischemia-reperfusion injuries accompanied by retinal swelling, neuronal cell death and glial cell activation [4]–[6].

Cells have highly developed endogenous antioxidant defense systems to counteract the oxidative stress generated in many diseases [7], [8]. Antioxidant/electrophile response element (ARE/EpRE)-regulated phase II detoxifying enzymes and antioxidants is one of the major antioxidant pathways involved in counteracting increased oxidative stress and maintaining the redox status in many tissues [7], [9]. Heme oxygenase-1 (HO-1), the rate-limiting enzyme that catalyzes the degradation of heme to biliverdin, carbon oxide (CO) and iron, is one of the ARE-regulated phase II detoxifying enzymes and antioxidants, which are regulated by the redox-sensitive transcription factor nuclear factor erythroid 2-related factor (Nrf2) [10]. Nrf2 demonstrates a protective role against neuronal and vascular degeneration in retinal ischemia-reperfusion injury [11]. HO-1 has also been reported to have the most AREs on its promoter, making it a highly effective therapeutic target for protection against neurodegenerative diseases [12]. Overexpression of HO-1 is neuroprotective in a model of permanent middle cerebral artery occlusion (MCAO) in transgenic mice [13]. Furthermore, pharmacological induction of HO-1 has been shown to protect the retina from acute glaucoma-induced ischemia-reperfusion injury [14].

Lycium barbarum polysaccharides (LBP) is the liquid fraction of the Lycium barbarum berries (Wolfberry), a traditional Chinese medicine with proposed anti-aging effects, extracted by a process involving the removal of the lipid soluble components, such as zeaxanthin and other carotenoids with alcohol [15]. Numerous studies have demonstrated the beneficial effects of LBP [16]–[19]. However, more recent studies have examined its protective effects in ocular diseases. LBP has been shown to protect retinal ganglion cells (RGCs) and retinal vasculature in several ocular disease models, including MCAO-induced retinal ischemia-reperfusion [20], [21]. Furthermore, lycium barbarum extracts protect the brain from blood-brain barrier disruption and cerebral edema in experimental stroke [17].

Although there have been many studies on the protective effects of LBP in various diseases, none of these studies have examined the contribution of the Nrf2/HO-1 antioxidant pathway. Considering the beneficial properties of LBP and the potential role of the Nrf2/HO-1 pathway, we used the acute glaucoma-induced ischemia-reperfusion model to analyze the mechanisms involved in the protective effects of LBP in this study. We hypothesized that the protection of LBP against retinal damage induced by ischemia-reperfusion injury occurs via activation of the Nrf2/HO-1 pathway.

## Materials and Methods

### Animals

Eight-week-old male Sprague-Dawley rats (300–350 g) were housed in a temperature-controlled room. The animals were maintained on a 12-hour light/12-hour dark schedule. Food and water were provided ad libitum. Full details of collection and sampling methods are described in appropriate sections below. At the end of the experiment, the animals were euthanized by an overdose of sodium pentobarbital. All of the experiments were performed in accordance with the Peking University guidelines for animal research, and the ARVO Statement for the Use of Animals in Ophthalmic and Vision Research. The experimental animal protocol used in this study was approved by the Peking University Institutional Animal Care and Use Committee (IACUC).

### Pre-treatment with LBP

Lycium barbarum (wolfberries) was purchased from a local supermarket from Ning Xia Huizu Autonomous Region, People’s Republic of China. Its dried fruit was ground into small pieces, delipidated and deproteinated in alcohol. LBP was then extracted by using 70°C hot water as described previously [21]. The extracts were freeze-dried into powder form for storage. For experimental use, the LBP solution was freshly prepared by dissolving the powder in phosphate-buffered saline (PBS; 0.01 M; pH 7.4). Rats were randomly assigned to the following groups: sham-operated group (eyes were cannulated with a 27-gauge infusion needle without the elevation of the saline reservoir, Control); vehicle group (animals were orally fed by gavage with PBS once daily for 1 week followed by 1-hour retinal ischemia, I/R); LBP+I/R group (animals were orally fed by gavage with LBP (1 mg/kg) once a day for 1 week followed by 1-hour retinal ischemia); and the LBP+I/R+ZnPP group (animals were orally fed by gavage with LBP (1 mg/kg) once a day for 1 week+intraperitoneal injection of ZnPP (the HO-1 inhibitor, 50 µmol/kg body weight, dissolved in equal amounts of PBS and 0.1 N NaOH, Sigma-Aldrich Corp., St. Louis, MO) 24 hours prior to ischemia followed by 1-hour retinal ischemia). Sulforaphane (SF), a specific Nrf2 inducer, was used as a positive control to induce the activation of the Nrf2/HO-1 antioxidant pathway in this study. In these experiments, SF (12.5 mg/kg, Toronto Research Chemicals Inc., North York, ON) was intraperitoneally administered 24 h prior to ischemia (SF+I/R). The animals were sacrificed with an overdose of sodium pentobarbital at 24 h or 7 days after ischemia.

### Ischemia-reperfusion Model and Experimental Protocol

The animals were anesthetized with a mixture of ketamine (80 mg/kg) and xylazine (8 mg/kg). The anterior chamber of the left eye was cannulated with a 27-gauge infusion needle that was connected to a physiological saline reservoir. The intraocular pressure was increased to 130 mm Hg for 60 minutes by elevation of the saline reservoir. Successful achievement of retinal ischemia was confirmed by the collapse of the central retinal artery and the whitening of the iris during the elevation of intraocular pressure [14].

### Detecting ROS Generation

The generation of retinal ROS was assessed using dihydroethidium (DHE; Invitrogen Molecular Probes, Eugene, OR) as previously described. Briefly, fresh retinas were harvested and quickly frozen in liquid nitrogen for cryosectioning (Leica CM1950; Leica Microsystems Ltd, Wetzlar, Germany). Cryosections (10 µm) were washed with a warm PBS solution and then incubated with 5 µM dihydroergotamine (DHE) in PBS for 30 minutes at 37°C. DHE specifically reacts with superoxide anions and is converted to the red fluorescent compound ethidium. The sections were examined and imaged using an inverted fluorescent microscope equipped with a digital camera (Eclipex Ti-S; Nikon Instech Co., Tokyo, Japan) under identical exposure conditions, and the optical densities of the staining in the outer nuclear layer (ONL), inner nuclear layer (INL), and ganglion cell layer (GCL) were measured from randomly selected images. Five measurements were obtained at 200 µm intervals using a commercial software program (Photoshop CS5; Adobe Corp., San Jose, CA).

### Immunohistochemistry

#### RNA binging protein with multiple splicing (RBPMS) antibody generation

A rabbit polyclonal antibody was generated against the N-terminus of the RNA Binding Protein Multiple Splice (RBPMS) polypeptide (RBPMS4-24), GGKAEKENTPSEANLQEEEVR by a commercial vendor (ProSci, Poway, CA). RBPMS is highly conserved among mammals and the polypeptide sequence used for immunization is identical in mouse, rat, monkey and human (NCBI Protein Bank, http://www.ncbi.nlm.nih.gov/protein). Rabbit sera were collected following immunization and affinity purified using a RBPMS polypeptide affinity column. The affinity purified antibody was shown to immunostain ganglion cells in mouse and rat retina (Rodriguez et al., 2013, submitted). To evaluate the specificity of the RBPMS immunostaining, a preabsorption control was performed with the rabbit antibody. Briefly, the RBPMS antibody was diluted in 0.1 M PB containing 0.5% Triton X-100 and mixed with the RBPMS polypeptide at a final concentration of 1 µg/ml for two hours at RT. No RBPMS immunostaining was present in tissue sections incubated with the rabbit antibody preabsorbed to RBPMS and processed by standard immunohistochemical techniques.

We used immunofluorescence to examine the localization and number of choline acetyltransferase (ChAT)-positive amacrine cells and RBPMS–positive ganglion cells in the retina. Localization and expression of HO-1 and Nrf2 were also examined using immunofluorecent staining. Apoptotic cells were stained using a TdT-mediated dUTPnick-end labeling (TUNEL)–based kit (Life Technologies, Grand Island, NY). Briefly, the eyes were enucleated, postfixed in 4% paraformaldehyde for 45 minutes, and embedded in OCT. Sections were transversely cut along the temporal-nasal axis of the eyeball. To ensure comparability, only sections that contained the optic nerve stump were used in this comparative study. Three retinal sections per animal were sampled to increase the reliability of the data, and the numbers obtained were pooled to obtain the final number of immunostained cells in each retina. The cryosections (10 µm) were thawed, air-dried, and washed three times with 0.01 M PBS (pH 7.4). Tissue specimens were first treated with 3% BSA (Sigma-Aldrich Corp., St. Louis, MO) in 0.3% Triton X-100 for 20 minutes at room temperature and then incubated with one of the following primary antibodies: goat polyclonal antibody against ChAT (Millipore Corp, Billerica, MA), rabbit polyclonal antibody against RBPMS, rabbit polyclonal antibody against HO-1 (Stressgen, Inc., San Diego, CA) or rabbit polyclonal antibody against Nrf2 (Santa Cruz Biotech Inc. Dallas, TX). Immunoreactivity was detected using a FITC-labeled secondary antibody (Abcam Inc., Cambridge, MA), and the cell nuclei were counterstained with 4′-6-diamidino-2-phenylindole (DAPI). The number of ChAT-, RBPMS- and TUNEL-positive cells in both the GCL and INL, and cells with Nrf2 nuclear accumulation in the GCL was quantified, respectively, in each section under a fluorescent microscope. For quantification of Nrf2, images of Nrf2 staining (green) and DAPI staining (blue) of the same area were merged together to locate the cells with nuclear Nrf2 accumulation. The color of DAPI staining was converted to red using a commercial software program (Photoshop CS5; Adobe Corp., San Jose, CA) before merging.

### Western Blotting Analyses

The eyes were enucleated, and the retinas were collected and flash-frozen at −80°C within 2 minutes of enucleation. The retinas were subsequently ultrasonically homogenized at 4°C in 300 mL RIPA buffer containing 50 mM Tris (pH 7.4), 150 mM NaCl, 10 mM EDTA, 0.1% SDS, 1% NP-40, 0.5% sodium deoxycholate, 1 mM Na_3_VO_4_, 1 mM NaF, 1 mM EGTA, 1 mM phenylmethylsulfonyl fluoride, and proteinase inhibitors. The protein concentrations were determined using a BCA protein assay to ensure equal protein loading, and 20 µg of protein in each lane were separated by 10% or 12% SDS-PAGE. Next, the proteins were transferred onto a nitrocellulose membrane (Millipore Corp, Billerica, MA) and then blocked and probed with either rabbit polyclonal anti–HO-1 (Stressgen Biotech Inc, Philadelphia, PA.) antibody or goat polyclonal antibody against ChAT (Millipore Corp, Billerica, MA). A peroxidase-conjugated anti–rabbit secondary antibody (PerkinElmer, Inc., Wellesley, MA) was used, and the blots were also probed for β-actin (Sigma-Aldrich Corp.) as a loading control. The protein bands were visualized using the ECL Western blotting detection reagent (GE Healthcare Life Science, Uppsala, Sweden) according to the manufacturer’s instructions. For quantification, blots from at least five independent experiments (5 animals per group) were quantified using Image J software.

### Statistical Analysis

The data were expressed as the means ± SEM. Analysis between multiple groups was performed using one-way ANOVA analysis followed by Bonferroni multiple comparison post-tests. P<0.05 was considered statistically significant.

## Results

### LBP Protected Retinal Cells against Apoptosis after I/R

**Figure 1:**
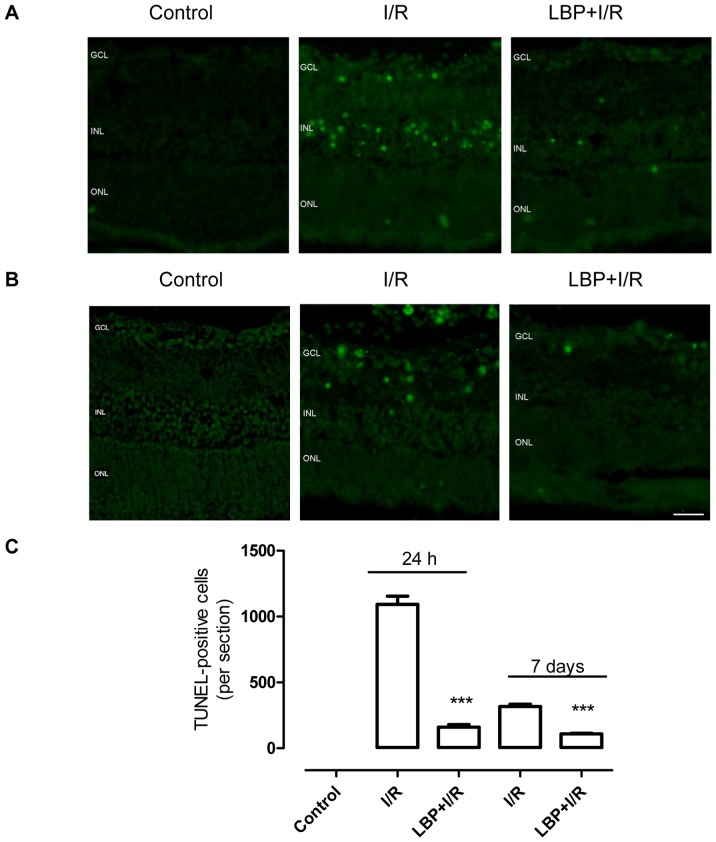
LBP inhibits ischemia-reperfusion-induced retinal cell apoptosis. Apoptotic cells in the I/R retina were stained using a TUNEL-kit as previously described in the Methods section. **A, B:** Representative micrographs of TUNEL-stained retinal sections obtained at 24 h (**A**) or 7 days (**B**) after ischemia. **C,** Quantitative analysis of TUNEL-positive cells in the retina (mean ± SEM, n = 5). Control: sham-operated animal, I/R: vehicle-treated animal with 1 h ischemia, and LBP+I/R: LBP-pretreated animal with 1 h ischemia. ***p<0.001 compared to I/R at the same time point. Scale bar: 20 µm. GCL: ganglion cell layer; INL: inner nuclear layer; ONL: outer nuclear layer.

As shown in Figure 1, ischemia for 1 h and reperfusion for 24 h (Fig. 1 A) or 7 days (Fig. 1 B) resulted in significant increases in the number of TUNEL-positive cells in the retina, predominantly in the INL and GCL, indicating that ischemia/reperfusion results in cell apoptosis in the retina. Significantly less TUNEL-positive cells were found in the INL and GCL in LBP-pretreated retinas when compared to vehicle-treated retinas at both 24 h and 7 days after I/R, suggesting that pretreatment with LBP (1 mg/kg body weight) for 1 wk significantly protected retinal cells against I/R-induced damage. Moreover, these protective effects persisted for at least 7 days.

### LBP Protected Retinal Ganglion Cells from I/R Damage

**Figure 2:**
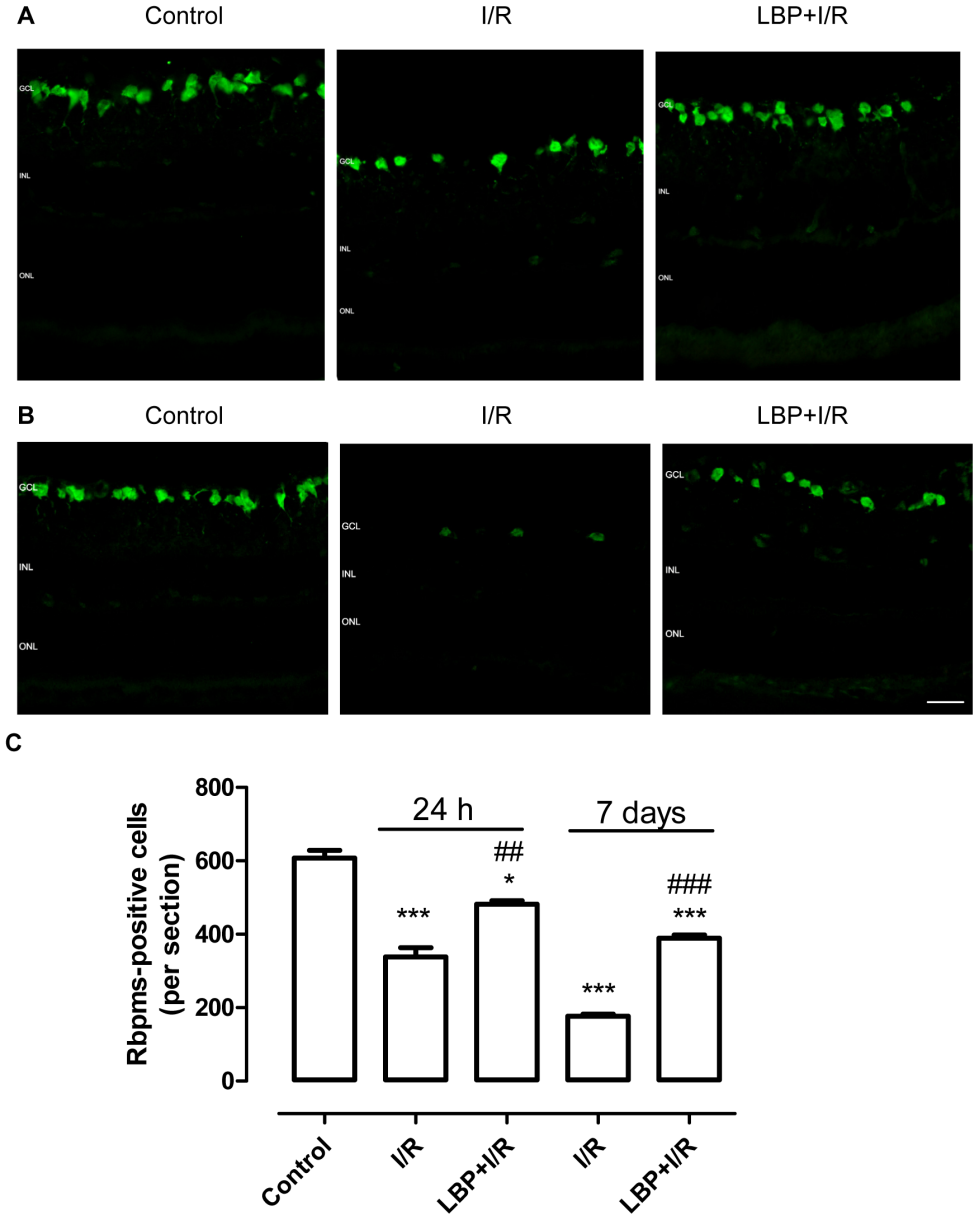
LBP protects retinal ganglion cells from ischemia-reperfusion-induced damage. Retinal ganglion cells were stained with a specific retinal ganglion cell marker, Rbpms. A, B: Representative micrographs of Rbpms-stained retinal sections obtained at 24 h (**A**) or 7 days (**B**) after ischemia. **C,** Quantitative analysis of Rbpms-positive cells in the retinal ganglion cell layer (mean ± SEM, n = 5). Control: sham-operated animal, I/R: vehicle-treated animal with 1 h ischemia, and LBP+I/R: LBP-pretreated animal with 1 h ischemia. *p<0.05, ***p<0.001 compared to control, ## *p*<0.01, ### *p*<0.001 compared to I/R at the same time point. Scale bar: 20 µm. GCL: ganglion cell layer; INL: inner nuclear layer; ONL: outer nuclear layer.

To further demonstrate whether pretreatment of LBP exhibited protective effects on retinal ganglion cells after I/R-induced damage, a specific marker of RGCs, RBPMS [22], [23], was used in this study. As shown in Figure 2, ischemia for 1 h and reperfusion for 24 h resulted in a nearly 50% decrease in the number of RGCs. At 7 days after ischemia, only 30% of RGCs remained in the retina. However, in the LBP-pretreated animals, the rate of RGC loss was delayed. In addition, more than 50% of the RGCs were in the retina 7 days after the ischemic insult.

### LBP Protected Retinal Amacrine Cells from I/R Damage

**Figure 3:**
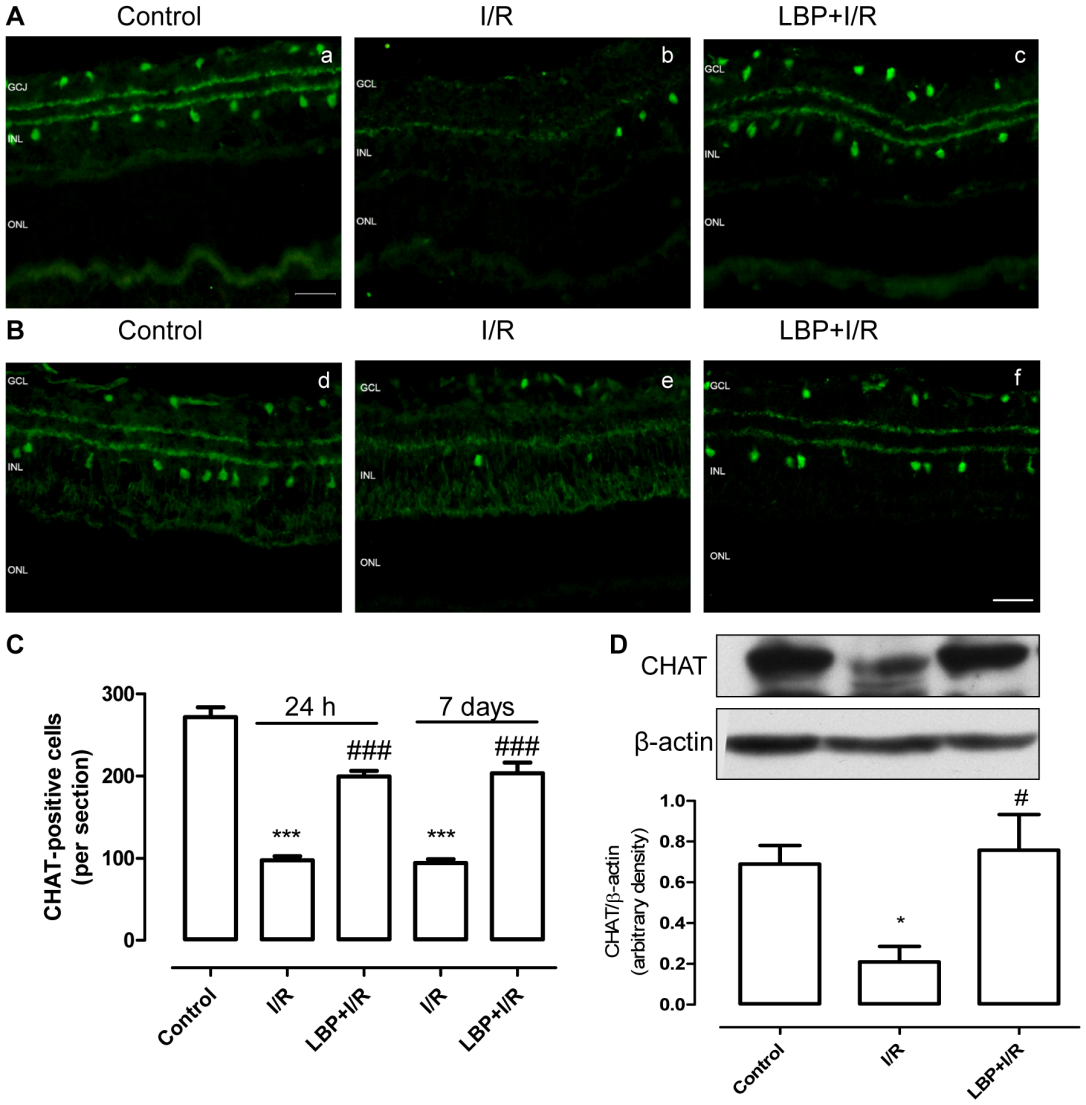
LBP protects retinal amacrine cells against ischemia-reperfusion-induced damage. Retinal amacrine cells were stained with anti-ChAT antibody. **A, B**: Representative micrographs of retinal sections stained with anti-ChAT antibody at 24 h (**A**) or 7 days (**B**) after ischemia. **C:** Quantitative analysis of ChAT-positive cells in the GCL and INL (mean ± SEM, n = 5). **D, E:** Representative immunoblot of ChAT levels in whole retina (upper panel) at 24 h (**D**) or 7 days (**E**) after ischemia and densitometric analysis of ChAT expression relative to loading control (lower panel) (mean ± SEM, n = 5). Control: sham-operated animal, I/R: vehicle-treated animal with 1 h ischemia, and LBP+I/R: LBP-pretreated animal with 1 h ischemia. *p<0.05, ***p<0.001 compared to control, # *p*<0.05, ### *p*<0.001 compared to I/R at the same time point. Scale bar: 20 µm. GCL: ganglion cell layer; INL: inner nuclear layer; ONL: outer nuclear layer.

A choline acetyltransferase (ChAT) antibody was used as a marker for cholinergic neurons in the retina. As shown in Figure 3A, in the non-ischemic control retina, ChAT-positive amacrine cells were present in the GCL and innermost layer of the INL. At 24 h after I/R, the number of ChAT-positive cells in the two cellular layers in the vehicle-treated I/R retina was much less compared to the non-ischemic control retina. Conversely, when compared with the vehicle-treated I/R retina, the LBP-treated I/R retina had an increase in the number of ChAT-positive cells. Similar results were observed in the retina 7 days after I/R (Figure 3B). These results were further confirmed by an immunoblotting study. As shown in Figures 3D and 3E, the ChAT protein levels in vehicle-treated I/R retinas were significantly less compared to the non-I/R retina, and LBP-pretreatment significantly upregulated ChAT levels in the retina after I/R.

### LBP Downregulated ROS Generation in I/R Retinas

**Figure 4:**
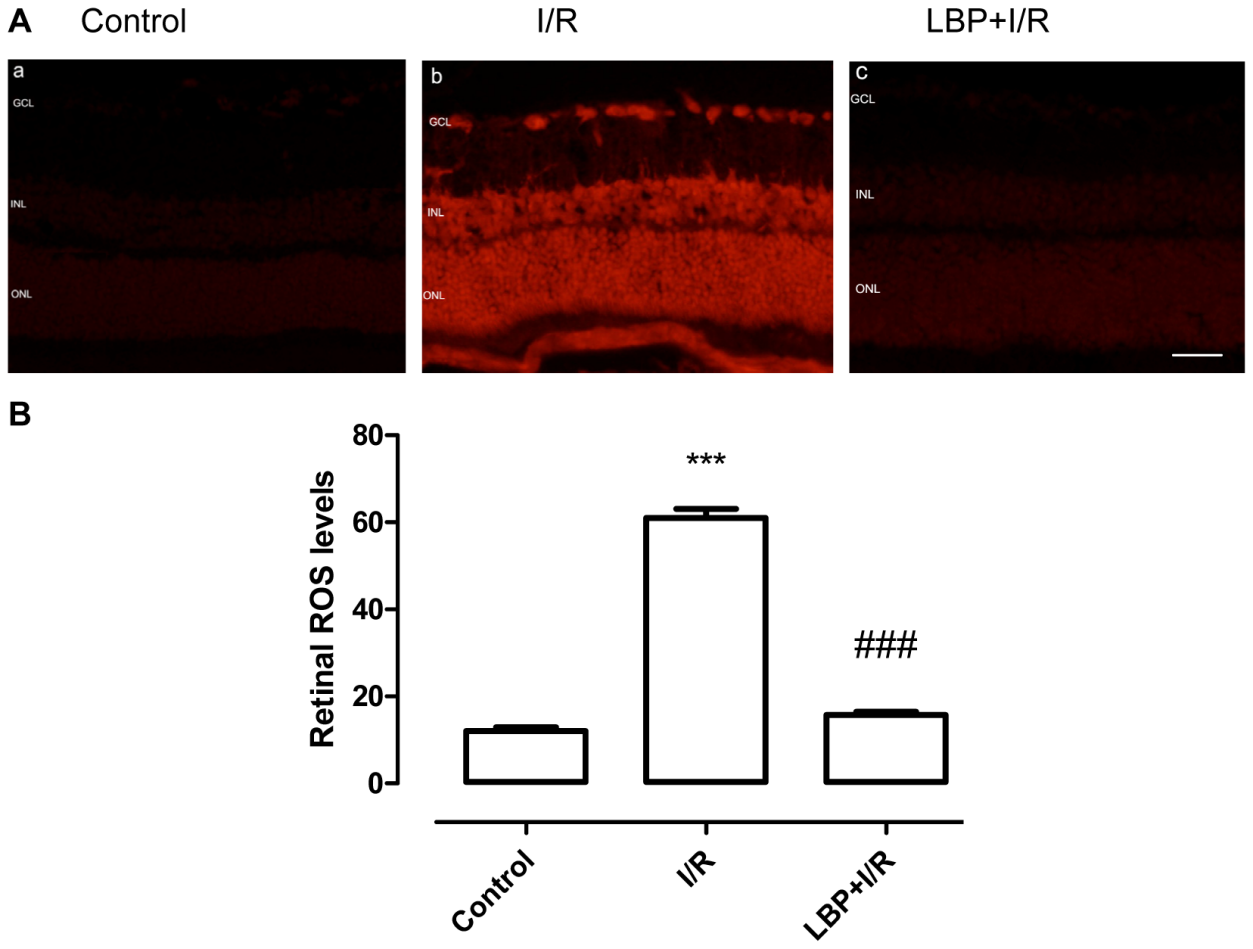
LBP reduces ROS generation in the retina after ischemia-reperfusion. ROS generation in fresh retinas was detected using DHE staining. (**A**) Representative micrographs of retinal sections stained with DHE (24 h after ischemia). (**B**) Quantitative analysis of ROS levels in the whole retina. The fluorescent intensities of the DHE-labeled neurons were quantified using an image analysis software program (Adobe Photoshop CS5; Adobe Corp.) (mean ± SEM, n = 5). ***p<0.001 compared to control, ### *p*<0.001 compared to I/R. Scale bar: 20 µm. Control: sham-operated animal, I/R: vehicle-treated animal with 1 h ischemia, and LBP+I/R: LBP-pretreated animal with 1 h ischemia. GCL: ganglion cell layer; INL: inner nuclear layer; ONL: outer nuclear layer.

The generation of ROS in fresh retinas was detected using DHE staining. As shown in Fig. 4, the basal level of ROS in non-ischemic control retinas was low (Fig. 4A–a). However, after 1 h of ischemia followed by 24 hrs of reperfusion, there was a dramatic increase in ROS generation in the entire retina (Fig. 4A–b). This effect was significantly decreased with LBP pretreatment (Fig. 4 A–c).

### LBP Upregulated the Nuclear Translocation of Nrf2 in RGCs

**Figure 5:**
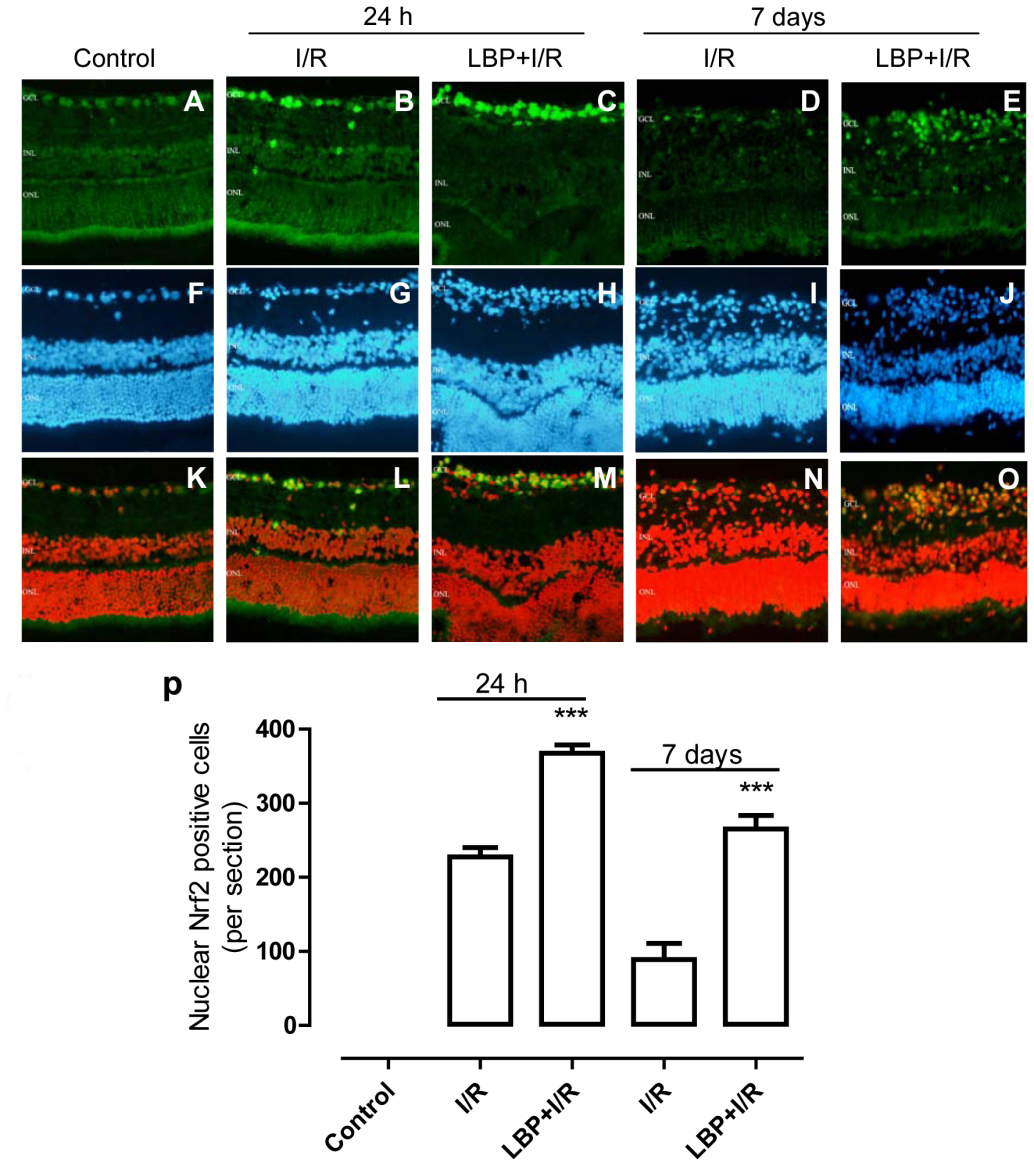
LBP enhanced nuclear Nrf2 accumulation in the retina after ischemia-reperfusion. The localization and expression of Nrf2 was determined by immunofluorescent staining using a specific anti-Nrf2 antibody (green). DAPI was used to counterstain the nucleus (blue). A–E: Representative micrographs of retinal sections stained with anti-Nrf2 at 24 h or 7 days after ischemia with or without LBP-pretreatment. F–J: Nucleus of cells shown in panel A-E were counterstained with DAPI; K–O: Merged images of A-E and F-J with the color of DAPI converted to red by Photoshop as described in the Methods; P: Quantitative analysis of nuclear Nrf2-positive cells in the retinal ganglion cell layer (mean ± SEM, n = 5). Control: sham-operated animal, I/R: vehicle-treated animal with 1 h ischemia, and LBP+I/R: LBP-pretreated animal with 1 h ischemia. ***p<0.001 compared to control, ### p<0.001 compared to I/R at the same time point. Scale bar: 20 µm. GCL: ganglion cell layer; INL: inner nuclear layer; ONL: outer nuclear layer.

In non-ischemic retinas, Nrf2 was diffusely displaced in the cytosol and nuclei of cells (Fig. 5 A). In vehicle-treated retinas at 24 h after I/R insult, retinal cells, especially cells in the GCL, displayed increased nuclear accumulation of Nrf2 as indicated by an increase in immunointensity of nuclear Nrf2 (Fig. 5 B). Moreover, LBP pretreatment further increased Nrf2 nuclear accumulation in the GCL (Fig. 5 C). At 7 days after I/R insult, I/R-induced Nrf2 nuclear accumulation was nearly diminished because very few cells were found with Nrf2 nuclear accumulation in the vehicle-treated I/R retina (Fig. 5 E). However, in the LBP-pretreated retina, cells with Nrf2 nuclear accumulation could still be found in the GCL (Fig. 5 F). Quantification analyses indicated that LBP pretreatment significantly increased the number of cells with accumulated nuclear Nrf2 in the GCL in retinas at 24 h and 7 days after I/R insult (Fig. 5 P). Furthermore, a specific Nrf2 activator, sulforaphane, was used to compare the effectiveness of LBP on Nrf2 activation. The effect of LBP on Nrf2 activation was similar to that of sulforaphane (data not shown).

### LBP Upregulated the Expression of HO-1 in the Retina

**Figure 6:**
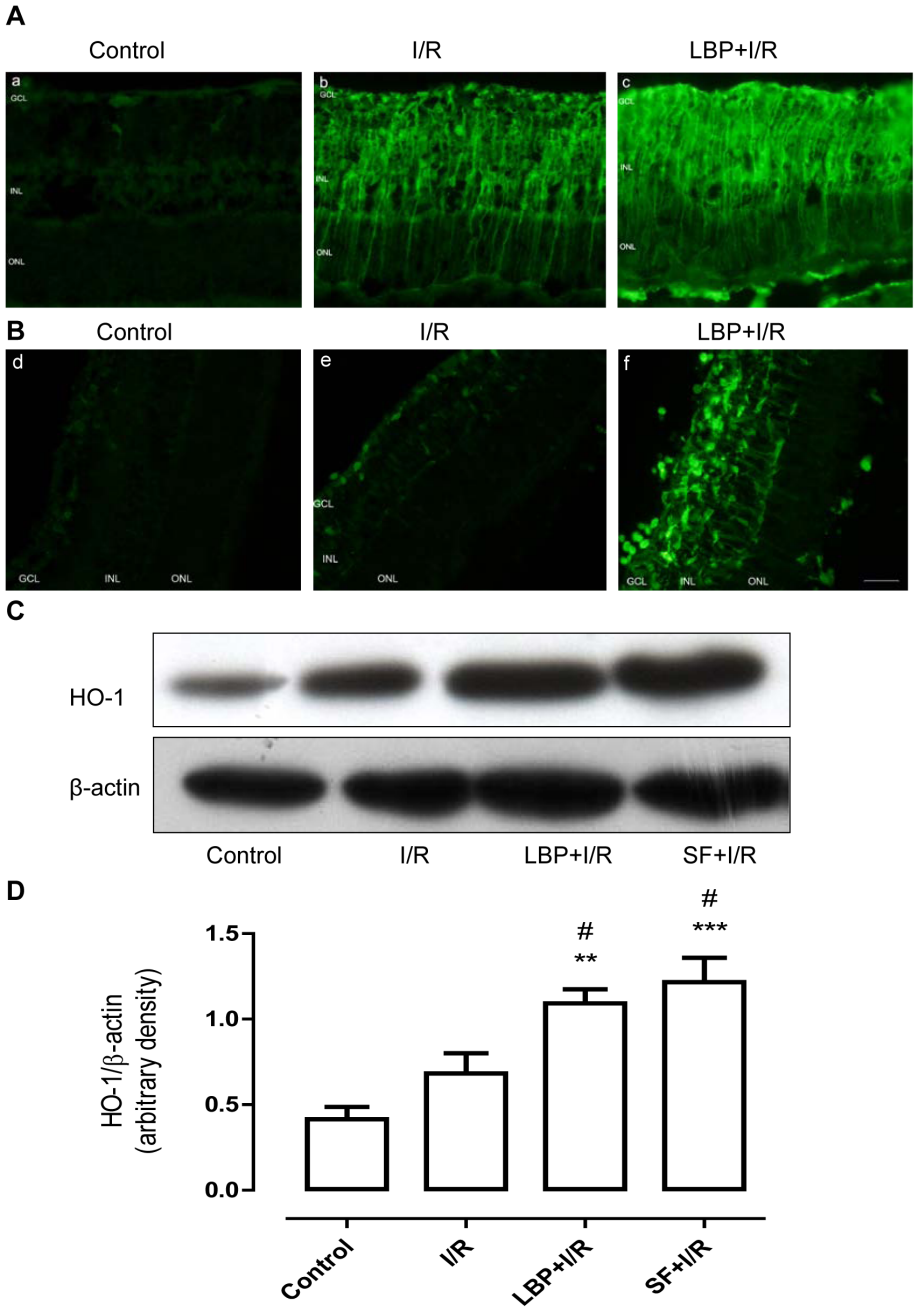
LBP upregulates the expression of HO-1 in the retina after ischemia-reperfusion. HO-1 expression in the retina was determined using immunofluorescent staining and immunoblotting. **A, B:** Representative micrographs of retinal sections stained with anti-HO-1 antibody at 24 h (A) or 7 days after ischemia. **C, D:** Representative immunoblot of HO-1 protein levels in whole retina at 24 h (**C**) and 7 days (**D**) after ischemia (upper panel) and densitometric analysis of HO-1 expression relative to the loading control (lower panel, mean ± SEM, n = 5). Control: sham-operated animal, I/R: vehicle-treated animal with 1 h ischemia, LBP+I/R: LBP-pretreated animal with 1 h ischemia, SF+I/R: sulforaphane-pretreated animal with 1 h ischemia. **p<0.01, ***p<0.001 compared to control, # *p*<0.05 compared to I/R. Scale bar: 20 µm. GCL: ganglion cell layer; INL: inner nuclear layer; ONL: outer nuclear layer.

Nrf2 is one of the transcription factors that regulate the expression of HO-1. Because LBP pretreatment induced an increase in the number of cells with nuclear accumulated Nrf2, the expression of HO-1, a downstream target gene of Nrf2, was examined using immunofluorescent staining and Western blotting analysis. As shown in Figure 6, non-ischemic control retinas have a relatively low HO-1 immunoreactivity (Fig. 6 A–a). In addition, 24 h after an ischemia insult, I/R induced robust immunoreactivity of HO-1 in the retina (Fig. 6 A–b). Pretreatment of LBP further enhanced HO-1 immunoreactivity in the retina after I/R (Fig. 6 A–c). These results were further confirmed by immunoblotting. As shown in Figure 6 C, the basal levels of HO-1 in the non-ischemic retina was low. However, I/R could induce an increase in the expression of HO-1 in the retina, although this increase failed to reach statistical significance. Moreover, LBP pretreatment significantly enhanced I/R-induced HO-1 expression. At 7 days after I/R insult, HO-1 immunoreactivity in the vehicle-treated I/R retina was nearly diminished or had returned to basal levels, whereas in the LBP-pretreated I/R retina, HO-1 immunoreactivity was still strong in the whole retina (Figure 6 B). Immunoblotting study has also revealed similar findings (Fig. 6 D). Similar to the Nrf2 studies, sulforaphane was also used as a positive control in the HO-1 experiments. The effect of LBP on the activation of HO-1 was similar to that of sulforaphane (Fig. 6 C).

### Inhibition of HO-1 Activity Abolished the LBP-induced Protective Effects in the Retina after I/R

**Figure 7:**
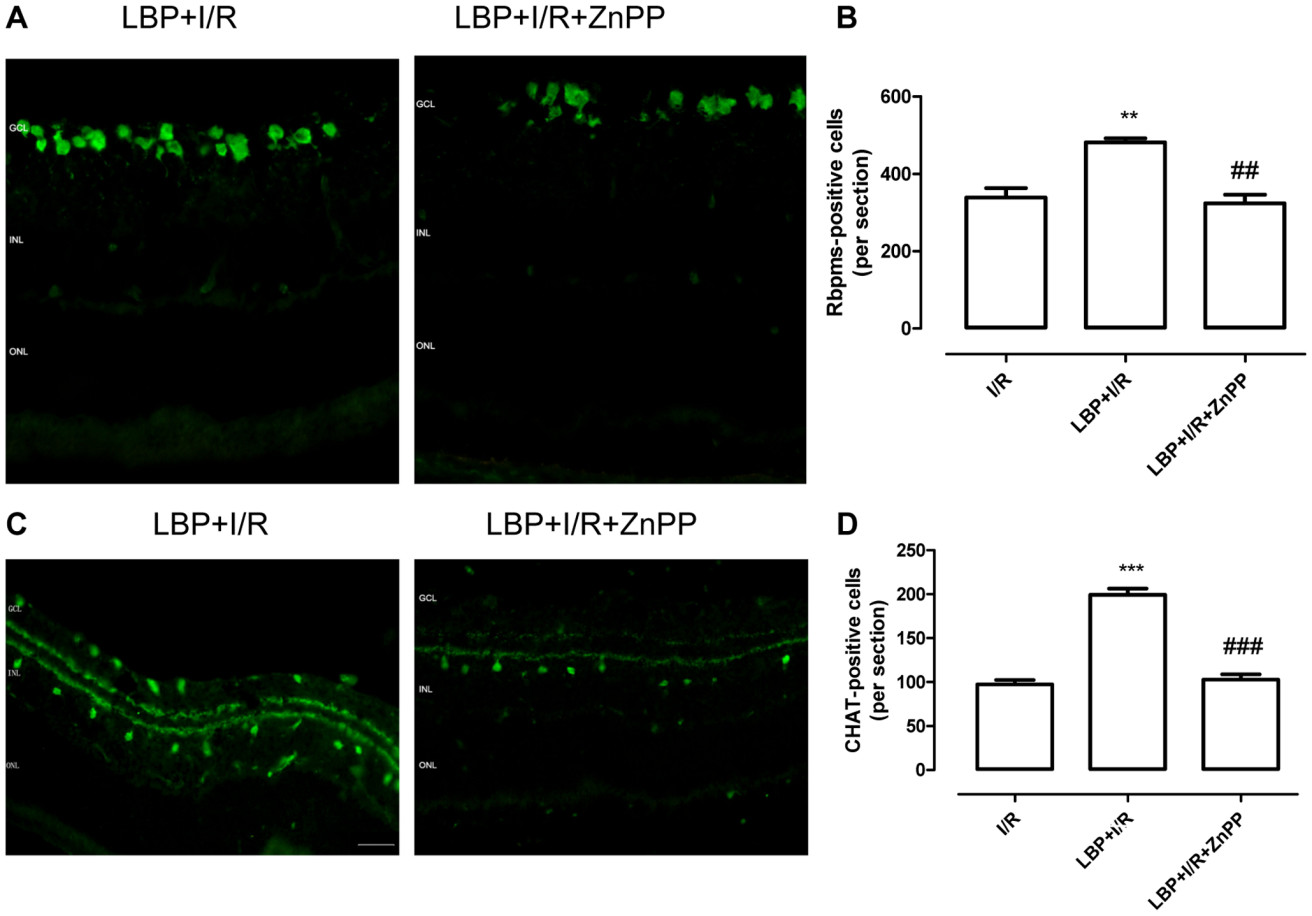
Abolishment of the protective effects of LBP after ischemia-reperfusion by the HO-1 inhibitor in retina. The involvement of HO-1 in the protective effects of LBP after ischemia-reperfusion in the retina was determined using the specific HO-1 inhibitor, ZnPP. **A**: Representative micrographs of retina sections stained with the RGC specific marker, Rbpms at 24 h after ischemia **B**: Quantitative analysis of Rbpms-positive cells in the retinal ganglion cell layer (mean ± SEM, n = 5). **C**: Representative micrographs of retina sections stained with the amacrine specific marker anti-ChAT at 24 h after ischemia. D: Quantitative analysis of ChAT-positive cells in the GCL and INL (mean ± SEM, n = 5). I/R: vehicle-treated animal with 1 h ischemia, LBP+I/R: LBP-pretreated animal with 1 h ischemia, LBP+I/R+ZnPP: LBP-pretreated animal with ZnPP injection 24 h before 1 h ischemia. **p<0.01, ***p<0.001 compared to I/R, ## *p*<0.01, ### *p*<0.001 compared to LBP+I/R. Scale bar: 20 µm. GCL: ganglion cell layer; INL: inner nuclear layer; ONL: outer nuclear layer.

To further investigate whether the protective effects of LBP on ganglion and amacrine cells in the I/R retina were mediated via activation of the Nrf2/HO-1 antioxidant pathway, a specific inhibitor of HO-1, ZnPP[14], was used. As shown in Figure 7, when pretreated with ZnPP, the number of RBPMS- and ChAT-positive cells in the LBP-pretreated I/R retina was decreased to levels similar to that observed in the vehicle-treated I/R retina.

## Discussion

The present study investigated the effects of LBP on retinal cells and the activation of the Nrf2/HO-1 antioxidant pathway in I/R retinas. Our data demonstrated that I/R induction enhanced ROS generation and increased the apoptosis of ganglion and amacrine cells in the retina. The Nrf2/HO-1 antioxidant pathway was adaptively activated to counteract the I/R-induced damage in the retina. LBP pretreatment did not only reduce the generation of ROS, but it also enhanced the activation of the Nrf2/HO-1 antioxidant pathway in I/R retinas. Furthermore, inhibition of HO-1 activity significantly blocked LBP-induced protective effects on I/R retinas, suggesting that the protective effects of LBP in I/R retinas was mediated, at least partly, by the activation of the Nrf2/HO-1 antioxidant pathway. Despite numerous studies on the protective effects of LBP in various diseases, to the best of our knowledge, our study is the first to demonstrate the contribution of the Nrf2/HO-1 antioxidant pathway to the protective effects of LBP in the I/R retina.

High intraocular pressure–induced retinal ischemia is a frequently used model for retinal ischemic studies [14], [21], [24]. This method produces global ischemia via the obstruction of both retinal and choroidal circulation, contributing to pathological features that are nearly identical to those observed in patients after a central retinal artery occlusion or ophthalmic artery occlusion. However, this model may also represent acute angle-closure glaucoma. Neuronal cell death, glial cell activation, retinal swelling, and oxidative injury are complications found in retinal ischemia-reperfusion injuries [14], [20].

Lycium barbarum is a dried fruit that is used as a food or medicine according to Chinese tradition [25]. Chemical composition analyses demonstrated that LBP consisted of several monosaccharides, namely glucose, fructose and xylose [26], [27]. It has been proposed that LBP provides anti-aging [28], anti-tumor [29], [30], cytoprotective [15], and neural modulatory [31] effects. Furthermore, LBP can reduce exercise-induced oxidative damage by decreasing plasma MDA formation and increasing SOD and GPx activity [19], [32]. It has also been shown that LBP can also protect multiple tissues against oxidative damage in streptozotocin-induced diabetic rats [32], [33] and in aged mice [28]. In mice, LBP protected the liver from carbon tetrachloride-induced oxidative stress and necroinflammation [34]. As an antioxidant, the cardioprotective effect of LBP has been demonstrated in acute doxorubicin-induced cardiotoxicity in beagle dogs [35] and in rats with cardiac ischemia-reperfusion injury [26]. Moreover, LBP also enhanced the levels of in vivo antioxidant biomarkers in the serum of healthy adults [16]. The protective effects of LBP against ocular diseases have also been recently demonstrated. Pre-treatment with LBP for 1 week effectively protected the retina against neuronal death, apoptosis, glial cell activation, aquaporin water channel upregulation, disruption of the blood-retina barrier and oxidative stress in MCAO and acute ocular hypertension-induced I/R retinas [20], [21]. Consistent with these results, the present study revealed that LBP pretreatment directly attenuated ROS generation and reduced retinal ganglion cell and amacrine cell apoptosis after I/R.

Heme oxygenase-1 (HO-1) is a rate-limiting enzyme, which catalyzes the degradation of heme into carbon monoxide, biliverdin, and ferritin [36]. The regulation of HO-1 gene expression occurs on multiple levels and is inducer-specific [37]–[39]. At the transcriptional level, HO-1 is mediated by the transcription factor Nrf2 [9], [11]. Under physiological conditions, Nrf2 is sequestered in the cytosol by Keap1 and is targeted for proteasomal degradation [40], [41]. In the presence of electrophiles or ROS, Nrf2 is released from Keap1 and then translocates into the nucleus, activating the transcription of target genes, including HO-1. As shown in this study, I/R injury resulted in a dramatic increase in ROS generation in the retina. Consequently, nuclear Nrf2 was increasingly accumulated and endogenous HO-1 was upregulated in the retinas after ischemia-reperfusion injury. However, this adaptive activation was temporary and had diminished at 7 days after ischemia-reperfusion injury. Pretreatment of LBP significantly enhanced and prolonged the activation of the Nrf2/HO-1 antioxidant pathway for up to 7 days after I/R injury and consequently protected retinal ganglion cells and amacrine cells against I/R-induced damage. In addition to LBP, many other neuronal protective agents, such as flavonoid [42], [43], reservatol (which is rich in polyphenol [44]), and sulforaphane have shown beneficial effects in the treatment of I/R-related neuronal diseases [45], [46] via the activation of the Nrf2/HO-1 antioxidant pathway. Studies using transgenic mice have also shown that pharmacological stimulation of HO-1 activity may ameliorate ischemic injury during the acute period of stroke [13], and pharmaceutical induction of HO-1 by the HO-1 activator CoPP ameliorated retinal damage due to I/R injury [14], [47], [48]. The most recent study by Varga et al demonstrated that activation of HO-1 was also involved in alpha-melanocyte-stimulating hormone (α-MSH)-induced protection of I/R retina [49], [50]. The cytoprotective properties of HO-1 are due to the by-products of HO-1-catalyzed heme cleavage, i.e., iron, bilirubin, and CO. Biliverdin and bilirubin are potent antioxidants, which scavenge peroxy radicals and inhibit lipid peroxidation [51]. CO shares some properties with NO, such as its effects on intracellular signaling processes, including anti-inflammatory, anti-proliferative, anti-apoptotic, and anti-coagulative effects [52]. The anti-inflammatory and anti-apoptotic effects of CO can reduce macrophage recruitment as well as ganglion and amacrine cell apoptosis as demonstrated in the present study after LBP pretreatment. Previous studies have also shown that pharmaceutical induction of HO-1 reduced macrophage recruitment and retinal cell apoptosis. Furthermore, enhancement of HO-1 expression after ischemia may extend neuronal survival [14]. In the present study, we demonstrated that pretreatment of LBP for 1 wk significantly enhanced Nrf2 nuclear accumulation and HO-1 expression after I/R injury. Concurrently, macrophage recruitment, and ganglion cell and amacrine cell apoptosis were also significantly inhibited by LBP pretreatment. Taken together, these results suggested that activation of the Nrf2/HO-1 antioxidant pathway contributed to the protective effects of LBP in the retina after I/R injury. Moreover, the involvement of HO-1 in the beneficial effects of LBP in the retina was further demonstrated using the HO-1 inhibitor ZnPP. As shown in the present study, ZnPP treatment significantly blocked the LBP-induced protective effects in the retina, including the prevention of macrophage recruitment and inhibition of ganglion cell and amacrine cell apoptosis after I/R injury. Taken together, our study demonstrated that the Nrf2/HO-1 antioxidant pathway contributes to the protective effects of LBP in the rodent retina after I/R-induced damage. Pretreatment with LBP may also strongly potentiate the cell’s adaptive antioxidant ability.

## References

[1] Zheng L, Gong B, Hatala DA, Kern TS (2007) Retinal ischemia and reperfusion causes capillary degeneration: similarities to diabetes. Invest Ophthalmol Vis Sci 48: 361–367. 17197555 10.1167/iovs.06-0510

[2] Osborne NN, Ugarte M, Chao M, Chidlow G, Bae JH, et al (1999) Neuroprotection in relation to retinal ischemia and relevance to glaucoma. Surv Ophthalmol 43 Suppl 1S102–S128. 10416754 10.1016/s0039-6257(99)00044-2

[3] Junk AK, Mammis A, Savitz SI, Singh M, Roth S, et al (2002) Erythropoietin administration protects retinal neurons from acute ischemia-reperfusion injury. Proc Natl Acad Sci U S A 99: 10659–10664. 12130665 10.1073/pnas.152321399 PMC125005

[4] Bonne C, Muller A, Villain M (1998) Free radicals in retinal ischemia. Gen Pharmacol 30: 275–280. 9510074 10.1016/s0306-3623(97)00357-1

[5] Muller A, Maurin L, Bonne C (1998) Free radicals and glutamate uptake in the retina. Gen Pharmacol 30: 315–318. 9510079 10.1016/s0306-3623(97)00362-5

[6] Shibuki H, Katai N, Yodoi J, Uchida K, Yoshimura N (2000) Lipid peroxidation and peroxynitrite in retinal ischemia-reperfusion injury. Invest Ophthalmol Vis Sci 41: 3607–3614. 11006259

[7] He M, Siow RC, Sugden D, Gao L, Cheng X, et al (2011) Induction of HO-1 and redox signaling in endothelial cells by advanced glycation end products: a role for Nrf2 in vascular protection in diabetes. Nutr Metab Cardiovasc Dis 21: 277–285. 20227863 10.1016/j.numecd.2009.12.008

[8] Tanito M, Masutani H, Kim YC, Nishikawa M, Ohira A, et al (2005) Sulforaphane induces thioredoxin through the antioxidant-responsive element and attenuates retinal light damage in mice. Invest Ophthalmol Vis Sci 46: 979–987. 15728556 10.1167/iovs.04-1120

[9] Siow RC, Ishii T, Mann GE (2007) Modulation of antioxidant gene expression by 4-hydroxynonenal: atheroprotective role of the Nrf2/ARE transcription pathway. Redox Rep 12: 11–15. 17263901 10.1179/135100007X162167

[10] Itoh K, Chiba T, Takahashi S, Ishii T, Igarashi K, et al (1997) An Nrf2/small Maf heterodimer mediates the induction of phase II detoxifying enzyme genes through antioxidant response elements. Biochem Biophys Res Commun 236: 313–322. 9240432 10.1006/bbrc.1997.6943

[11] Wei Y, Gong J, Yoshida T, Eberhart CG, Xu Z, et al (2011) Nrf2 has a protective role against neuronal and capillary degeneration in retinal ischemia-reperfusion injury. Free Radic Biol Med 51: 216–224. 21545836 10.1016/j.freeradbiomed.2011.04.026 PMC3997112

[12] Shah ZA, Li RC, Ahmad AS, Kensler TW, Yamamoto M, Biswal S, et al (2010) The flavanol (-)-epicatechin prevents stroke damage through the Nrf2/HO1 pathway. J Cereb Blood Flow Metab 30: 1951–1961. 20442725 10.1038/jcbfm.2010.53 PMC3002885

[13] Panahian N, Yoshiura M, Maines MD (1999) Overexpression of heme oxygenase-1 is neuroprotective in a model of permanent middle cerebral artery occlusion in transgenic mice. J Neurochem 72: 1187–1203. 10037492 10.1111/j.1471-4159.1999.721187.x

[14] Sun MH, Pang JH, Chen SL, Han WH, Ho TC, et al (2010) Retinal protection from acute glaucoma-induced ischemia-reperfusion injury through pharmacologic induction of heme oxygenase-1. Invest Ophthalmol Vis Sci 51: 4798–4808. 20357190 10.1167/iovs.09-4086

[15] Yu MS, Leung SK, Lai SW, Che CM, Zee SY, et al (2005) Neuroprotective effects of anti-aging oriental medicine Lycium barbarum against beta-amyloid peptide neurotoxicity. Exp Gerontol 40: 716–727. 16139464 10.1016/j.exger.2005.06.010

[16] Amagase H, Sun B, Borek C (2009) Lycium barbarum (goji) juice improves in vivo antioxidant biomarkers in serum of healthy adults. Nutr Res 29: 19–25. 19185773 10.1016/j.nutres.2008.11.005

[17] Yang D, Li SY, Yeung CM, Chang RC, So KF, et al (2012) Lycium barbarum extracts protect the brain from blood-brain barrier disruption and cerebral edema in experimental stroke. PLoS One 7: e33596. 22438957 10.1371/journal.pone.0033596 PMC3306421

[18] Chiu K, Chan HC, Yeung SC, Yuen WH, Zee SY, et al (2009) Modulation of microglia by Wolfberry on the survival of retinal ganglion cells in a rat ocular hypertension model. J Ocul Biol Dis Infor 2: 47–56. 19672466 10.1007/s12177-009-9023-9 PMC2723674

[19] Shan X, Zhou J, Ma T, Chai Q (2011) Lycium barbarum Polysaccharides Reduce Exercise-Induced Oxidative Stress. Int J Mol Sci 12: 1081–1088. 21541044 10.3390/ijms12021081 PMC3083691

[20] Li SY, Yang D, Yeung CM, Yu WY, Chang RC, et al (2011) Lycium barbarum polysaccharides reduce neuronal damage, blood-retinal barrier disruption and oxidative stress in retinal ischemia/reperfusion injury. PLoS One 6: e16380. 21298100 10.1371/journal.pone.0016380 PMC3027646

[21] Mi XS, Feng Q, Lo AC, Chang RC, Lin B, et al (2012) Protection of retinal ganglion cells and retinal vasculature by Lycium barbarum polysaccharides in a mouse model of acute ocular hypertension. PLoS One 7: e45469. 23094016 10.1371/journal.pone.0045469 PMC3477168

[22] Kwong JM, Quan A, Kyung H, Piri N, Caprioli J (2011) Quantitative analysis of retinal ganglion cell survival with Rbpms immunolabeling in animal models of optic neuropathies. Invest Ophthalmol Vis Sci 52: 9694–9702. 22110060 10.1167/iovs.11-7869 PMC3341125

[23] Hornberg H, Wollerton-van HF, Maurus D, Zwart M, Svoboda H, et al (2013) RNA-Binding Protein Hermes/RBPMS Inversely Affects Synapse Density and Axon Arbor Formation in Retinal Ganglion Cells In Vivo. J Neurosci 33: 10384–10395. 23785151 10.1523/JNEUROSCI.5858-12.2013 PMC4603358

[24] Goldblum D, Mittag T (2002) Prospects for relevant glaucoma models with retinal ganglion cell damage in the rodent eye. Vision Res 42: 471–478. 11853763 10.1016/s0042-6989(01)00194-8

[25] Chang RC, So KF (2008) Use of anti-aging herbal medicine, Lycium barbarum, against aging-associated diseases. What do we know so far? Cell Mol Neurobiol 28: 643–652. 17710531 10.1007/s10571-007-9181-x PMC11514989

[26] Lu SP, Zhao PT (2010) Chemical characterization of Lycium barbarum polysaccharides and their reducing myocardial injury in ischemia/reperfusion of rat heart. Int J Biol Macromol 47: 681–684. 20813126 10.1016/j.ijbiomac.2010.08.016

[27] Wu HT, He XJ, Hong YK, Ma T, Xu YP, et al (2010) Chemical characterization of Lycium barbarum polysaccharides and its inhibition against liver oxidative injury of high-fat mice. Int J Biol Macromol 46: 540–543. 20193709 10.1016/j.ijbiomac.2010.02.010

[28] Li XM, Ma YL, Liu XJ (2007) Effect of the Lycium barbarum polysaccharides on age-related oxidative stress in aged mice. J Ethnopharmacol 111: 504–511. 17224253 10.1016/j.jep.2006.12.024

[29] Mao F, Xiao B, Jiang Z, Zhao J, Huang X, et al (2011) Anticancer effect of Lycium barbarum polysaccharides on colon cancer cells involves G0/G1 phase arrest. Med Oncol 28: 121–126. 20066520 10.1007/s12032-009-9415-5

[30] Luo Q, Li Z, Yan J, Zhu F, Xu RJ, et al (2009) Lycium barbarum polysaccharides induce apoptosis in human prostate cancer cells and inhibits prostate cancer growth in a xenograft mouse model of human prostate cancer. J Med Food 12: 695–703. 19735167 10.1089/jmf.2008.1232

[31] Lau BW, Lee JC, Li Y, Fung SM, Sang YH, et al (2012) Polysaccharides from wolfberry prevents corticosterone-induced inhibition of sexual behavior and increases neurogenesis. PLoS One 7: e33374. 22523540 10.1371/journal.pone.0033374 PMC3327693

[32] Niu AJ, Wu JM, Yu DH, Wang R (2008) Protective effect of Lycium barbarum polysaccharides on oxidative damage in skeletal muscle of exhaustive exercise rats. Int J Biol Macromol 42: 447–449. 18405964 10.1016/j.ijbiomac.2008.02.003

[33] Li XM (2007) Protective effect of Lycium barbarum polysaccharides on streptozotocin-induced oxidative stress in rats. Int J Biol Macromol 40: 461–465. 17166579 10.1016/j.ijbiomac.2006.11.002

[34] Xiao J, Liong EC, Ching YP, Chang RC, So KF, et al (2012) Lycium barbarum polysaccharides protect mice liver from carbon tetrachloride-induced oxidative stress and necroinflammation. J Ethnopharmacol 139: 462–470. 22138659 10.1016/j.jep.2011.11.033

[35] Xin Y, Zhang S, Gu L, Liu S, Gao H, et al (2011) Electrocardiographic and biochemical evidence for the cardioprotective effect of antioxidants in acute doxorubicin-induced cardiotoxicity in the beagle dogs. Biol Pharm Bull 34: 1523–1526. 21963490 10.1248/bpb.34.1523

[36] Siow RC, Sato H, Mann GE (1999) Heme oxygenase-carbon monoxide signalling pathway in atherosclerosis: anti-atherogenic actions of bilirubin and carbon monoxide? Cardiovasc Res 41: 385–394. 10341838 10.1016/s0008-6363(98)00278-8

[37] Ryter SW, Alam J, Choi AM (2006) Heme oxygenase-1/carbon monoxide: from basic science to therapeutic applications. Physiol Rev 86: 583–650. 16601269 10.1152/physrev.00011.2005

[38] Alam J, Igarashi K, Immenschuh S, Shibahara S, Tyrrell RM (2004) Regulation of heme oxygenase-1 gene transcription: recent advances and highlights from the International Conference (Uppsala, 2003) on Heme Oxygenase. Antioxid Redox Signal 6: 924–933. 15345152 10.1089/ars.2004.6.924

[39] Martin D, Rojo AI, Salinas M, Diaz R, Gallardo G, et al (2004) Regulation of heme oxygenase-1 expression through the phosphatidylinositol 3-kinase/Akt pathway and the Nrf2 transcription factor in response to the antioxidant phytochemical carnosol. J Biol Chem 279: 8919–8929. 14688281 10.1074/jbc.M309660200

[40] Jeong WS, Jun M, Kong AN (2006) Nrf2: a potential molecular target for cancer chemoprevention by natural compounds. Antioxid Redox Signal 8: 99–106. 16487042 10.1089/ars.2006.8.99

[41] Cheng X, Siow RC, Mann GE (2011) Impaired redox signaling and antioxidant gene expression in endothelial cells in diabetes: a role for mitochondria and the nuclear factor-E2-related factor 2-Kelch-like ECH-associated protein 1 defense pathway. Antioxid Redox Signal 14: 469–487. 20524845 10.1089/ars.2010.3283

[42] Chao HM, Chuang MJ, Liu JH, Liu XQ, Ho LK, et al (2013) Baicalein Protects Against Retinal Ischemia by Antioxidation, Antiapoptosis, Downregulation of HIF-1alpha, VEGF, and MMP-9 and Upregulation of HO-1. J Ocul Pharmacol Ther 29: 539–549. 23537149 10.1089/jop.2012.0179 PMC3708628

[43] Szabo ME, Gallyas E, Bak I, Rakotovao A, Boucher F, et al (2004) Heme oxygenase-1-related carbon monoxide and flavonoids in ischemic/reperfused rat retina. Invest Ophthalmol Vis Sci 45: 3727–3732. 15452083 10.1167/iovs.03-1324

[44] Liu XQ, Wu BJ, Pan WH, Zhang XM, Liu JH, et al (2013) Resveratrol mitigates rat retinal ischemic injury: the roles of matrix metalloproteinase-9, inducible nitric oxide, and heme oxygenase-1. J Ocul Pharmacol Ther 29: 33–40. 23075401 10.1089/jop.2012.0141 PMC3552178

[45] Danilov CA, Chandrasekaran K, Racz J, Soane L, Zielke C, et al (2009) Sulforaphane protects astrocytes against oxidative stress and delayed death caused by oxygen and glucose deprivation. Glia 57: 645–656. 18942756 10.1002/glia.20793 PMC2657190

[46] Soane L, Li DW, Fiskum G, Bambrick LL (2010) Sulforaphane protects immature hippocampal neurons against death caused by exposure to hemin or to oxygen and glucose deprivation. J Neurosci Res 88: 1355–1363. 19998483 10.1002/jnr.22307 PMC3037525

[47] Peng PH, Chao HM, Juan SH, Chen CF, Liu JH, et al (2011) Pharmacological preconditioning by low dose cobalt protoporphyrin induces heme oxygenase-1 overexpression and alleviates retinal ischemia-reperfusion injury in rats. Curr Eye Res 36: 238–246. 21275512 10.3109/02713683.2010.539760

[48] rai-Gaun S, Katai N, Kikuchi T, Kurokawa T, Ohta K, et al (2004) Heme oxygenase-1 induced in muller cells plays a protective role in retinal ischemia-reperfusion injury in rats. Invest Ophthalmol Vis Sci 45: 4226–4232. 15505079 10.1167/iovs.04-0450

[49] Varga B, Szabadfi K, Kiss P, Fabian E, Tamas A, et al (2011) PACAP improves functional outcome in excitotoxic retinal lesion: an electroretinographic study. J Mol Neurosci 43: 44–50. 20567936 10.1007/s12031-010-9406-1

[50] Varga B, Gesztelyi R, Bombicz M, Haines D, Szabo AM, et al (2013) Protective Effect of Alpha-Melanocyte-Stimulating Hormone (alpha-MSH) on the Recovery of Ischemia/Reperfusion (I/R)-Induced Retinal Damage in A Rat Model. J Mol Neurosci 50: 558–570. 23504281 10.1007/s12031-013-9998-3 PMC3675276

[51] Ghattas MH, Chuang LT, Kappas A, Abraham NG (2002) Protective effect of HO-1 against oxidative stress in human hepatoma cell line (HepG2) is independent of telomerase enzyme activity. Int J Biochem Cell Biol 34: 1619–1628. 12379283 10.1016/s1357-2725(02)00097-3

[52] Ryter SW, Alam J, Choi AM (2006) Heme oxygenase-1/carbon monoxide: from basic science to therapeutic applications. Physiol Rev 86: 583–650. 16601269 10.1152/physrev.00011.2005

